# Socioeconomic position at the age of 30 and the later risk of a mental disorder: a nationwide population-based register study

**DOI:** 10.1136/jech-2022-219674

**Published:** 2023-02-06

**Authors:** Christian Hakulinen, Kaisla Komulainen, Kimmo Suokas, Sami Pirkola, Laura Pulkki-Råback, Sonja Lumme, Marko Elovainio, Petri Böckerman

**Affiliations:** 1 Department of Psychology and Logopedics, University of Helsinki, Helsinki, Finland; 2 Finnish Institute for Health and Welfare, Helsinki, Finland; 3 Faculty of Social Sciences, Tampere University, Tampere, Finland; 4 Research Program Unit, Faculty of Medicine, University of Helsinki, Helsinki, Finland; 5 School of Business and Economics, University of Jyväskylä, Jyväskylä, Finland; 6 Labour Institute for Economic Research LABORE, Helsinki, Finland; 7 IZA Institute of Labor Economics, Bonn, Germany

**Keywords:** EPIDEMIOLOGY, MENTAL HEALTH, SOCIAL CLASS, PSYCHIATRY

## Abstract

**Background:**

A study was undertaken to examine the association between multiple indicators of socioeconomic position (SEP) at the age of 30 and the subsequent risk of the most common mental disorders.

**Methods:**

All persons born in Finland between 1966 and 1986 who were alive and living in Finland at the end of the year when they turned 30 were included. Educational attainment, employment status and personal total income were used as the alternative measures of SEP. Cox proportional hazards models were used to examine the association of SEP at the age of 30 with later risk of mental disorders. Additional analyses were conducted using a sibling design to account for otherwise unobserved shared family characteristics. Competing risks models were used to estimate absolute risks.

**Results:**

The study population included 1 268 768 persons, 26% of whom were later diagnosed with a mental disorder. Lower SEP at age 30 was consistently associated with a higher risk of being later diagnosed with a mental disorder, even after accounting for shared family characteristics and prior history of a mental disorder. Diagnosis-specific analyses showed that the associations were considerably stronger when substance misuse or schizophrenia spectrum disorders were used as an outcome. Absolute risk analyses showed that, by the age of 52 years, 58% of persons who had low educational attainment at the age of 30 were later diagnosed with a mental disorder.

**Conclusions:**

Poor SEP at the age of 30 is associated with an increased risk of being later diagnosed with a mental disorder.

**WHAT IS ALREADY KNOWN ON THIS TOPIC**

- The association between socioeconomic position (SEP) and the incidence of mental disorders has been widely documented.
- The importance of different measures of SEP has rarely been compared in the same study.

**WHAT THIS STUDY ADDS**

- Lower SEP, and notably personal income, at the age of 30 was consistently associated with a higher risk of being later diagnosed with a mental disorder.
- These associations persisted even after accounting for shared family characteristics and a prior history of a mental disorder.
- The associations were considerably stronger when substance misuse or schizophrenia spectrum disorders were used as an outcome.
- By the age of 52 years, 58% of persons who had low educational attainment at the age of 30 were later diagnosed with a mental disorder.

**HOW THIS STUDY MIGHT AFFECT RESEARCH, PRACTICE OR POLICY**

- The burden of mental disorders is vastly greater among persons with low SEP, justifying public policies to tackle socioeconomic disparities.
- Policies that allocate greater amounts of preventive measures to persons with low SEP to mitigate the disease burden of mental disorders in the society should be encouraged.

## Introduction

Socioeconomic gradient in mental disorders has been well established.^1–7^ This association is known to be bidirectional, with low socioeconomic position (SEP) associated with future mental disorders,^8–10^ and mental disorders that have onset in childhood, adolescence or early adulthood followed by low SEP later in adulthood.^11–15^ Although most studies provide support for these associations, there is notable variability depending on the specific disorders examined; for example, whereas persons with substance misuse disorders or schizophrenia have a very low labour force participation rate,^12 13 16–18^ considerably higher rates have been reported among persons with mood or anxiety disorders.^11 13 15 19 20^

Most previous studies have focused on educational attainment or occupational status as the primary measures of SEP. Disposable income, which is a more direct measure of the available material resources at the individual and household level,^21^ has been used less frequently as it is often not as readily available to researchers as other measures of SEP. Although there are some notable exceptions,^22^ the importance and predictive power of different socioeconomic measures have rarely been examined and compared in the same study. Similarly, relatively few studies have been able to evaluate whether the association of SEP with later common mental disorders is explained by shared family characteristics that can have an influence on both SEP and the incidence of common mental disorders.

In the present study we used nationwide register-based data from Finland to conduct a comprehensive investigation of the association between SEP at the age of 30 and the subsequent risk of the most common major mental disorders—namely, substance misuse, schizophrenia spectrum, mood and anxiety disorders. We used three register-based measures for SEP (educational attainment, employment status and personal total income), and conducted the analyses also using a within-sibling research design to account for otherwise unobserved shared family characteristics that affect both SEP and the incidence of common mental disorders. To improve the applicability of the present findings in both research and public policy settings, we report both relative and absolute risks.^23^ Our study addressed the following research questions:

1. Are different socioeconomic measures—educational attainment, employment status and personal total income—assessed at the age of 30 associated with later risk of mental disorders?
2. To what degree are these associations diluted after accounting for unobserved family characteristics and the history of mental disorders before the age of 30?

## Methods

### Study population

We included all individuals born in Finland between 1966 and 1986 who were alive and living in Finland at the end of the year when they turned 30. Personal identifiers, which have been assigned to all Finnish citizens since 1969, were used to link data across several national registers. The ethics committee of the Finnish Institute of Health and Welfare (THL/730/6.02.01/2018) approved the study. Data were linked with the permission of Statistics Finland (TK-53-1696-16), the Finnish Institute of Health and Welfare and the Social Insurance Institution of Finland.

#### Measures of socioeconomic position (SEP) at the age of 30

The measures for SEP were obtained from the nationwide registers of Statistics Finland in the year the person turned 30. Employment status was assessed during the last week of each year. Individuals who were wage/salary earners or self-employed were classified as employed, persons who received unemployment benefits were classified as unemployed, and all others as being outside the labour force. Personal total (disposable) income was measured as the sum of wage and salary earnings, self-employment income, income transfers, social security benefits and capital income. To ensure the comparability of income over the observation period, the personal total income was deflated to the base year 2015 using the official consumer price index maintained by Statistics Finland. The highest completed education was classified as follows: 0=primary education (ie, only basic compulsory education); 1=secondary education (ie, completed upper secondary education or similar); 2=higher education (ie, completed at least a bachelor’s degree, a master’s degree, a polytechnic degree or similar).

#### Mental disorders

Information on mental disorders was obtained from the Finnish Care Register for Healthcare (FCR) maintained by the Finnish Institute for Health and Welfare and the sickness absence register (SAR) of the Social Insurance Institution of Finland. FCR contains information on all hospital admissions in Finland since 1969, secondary outpatient care since 1998, and primary care since 2011. SAR contains administrative information on sickness absences and diagnoses related to sickness absence spells that were granted due to a mental disorder and that lasted >9 days. The cut-off at 9 days is based on the Finnish sickness insurance system in which the employee is entitled to the normal full salary during the 9‐day waiting period. After that the employee is entitled to sickness allowance from the Social Insurance Institution of Finland (Kela). Data from SAR were available starting from 2004.

Mental disorders were diagnosed according to the International Statistical Classification of Diseases spectrum Health Problems, Tenth Revision (ICD-10) since 1995, according to the ICD-9 with DSM-III-R criteria from 1986 to 1995, and according to the ICD-8 from 1969 to 1986. In primary care, ICPC-2 International Classification of Primary Care is used in some facilities, and ICPC-2 mental health-related diagnoses were converted to corresponding ICD-10 sub-chapter categories.^24^ A description of the method used to account for partly overlapping register data entries in the FCR is publicly available.^25^

Mental disorders diagnosed after the age of 30 were categorised as follows: any mental disorder (F00–F99), substance misuse disorders (F10–19), schizophrenia spectrum disorders (F20–29), mood disorders (F30–39) and anxiety disorders (F40–48). Before the age of 30, only the diagnosis of any mental disorder (F00–F99) was used.

#### Covariates

Sex (0=men; 1=women), birth year, urbanicity (0=urban; 1=semi-urban; 2=rural) using the urban-rural classification system of the Finnish Environment Institute (SYKE), and the status of cohabitation (0=living alone; 1=cohabiting with someone) at the age of 30 were obtained from the registers compiled by Statistics of Finland.

### Statistical analyses

Persons were followed from the end of the year when they turned 30 until the first diagnosis of mental disorder, date of emigration from Finland, death or 31 December 2017, whichever came first. The maximum length of follow-up was thus 22 complete years, up to the age of 52 years for those born in 1966. The association of socioeconomic measures at the age of 30 with later mental disorders was examined using the Cox proportional hazards models, which was adjusted for sex, birth year, living alone and urbanicity. A further adjustment was made for the diagnosis of any mental disorder before the age of 30. To examine to what degree these associations were confounded by familial risk factors (ie, shared environmental and/or genetic factors), all analyses were repeated using stratified Cox proportional hazard models conducted within strata of full-sibling pairs.^26^ The results were reported as hazard ratios (ie, relative risks). Lastly, the cumulative incidence (absolute risk) of a mental disorder was estimated using competing risks regression,^27^ where emigration from Finland and death were treated as competing events. As sensitivity analyses, we ran all Cox proportional hazard models separately for mental disorder diagnoses retrieved from the FCR and SAR and the competing risks models also among persons without a mental disorder before the age of 30. All analyses were conducted using Stata version 16.1 (Stata Corp, College Station, Texas, USA).

### Role of the funding source

The funders of the study had no role in the study design, data collection, data analysis, data interpretation and writing of the manuscript. The corresponding author had full access to all study data and had final responsibility for the decision to submit for publication.

## Results

The study population included 1 268 768 persons who were followed for over 12.7 million person-years between 1996 and 2017. A total of 331 657 (26.14%) persons were diagnosed with a mental disorder over the follow-up period. Descriptive statistics of the study population are shown in table 1. Of the total population, 7% had a history of mental disorder treatment before the age of 30 years.

**Table 1:** Descriptive statistics of the study population (n=1 268 768)

	Whole study population	Without history of mental disorders	With history of mental disorders
	N (%) or mean (SD)	N (%) or mean (SD)	N (%) or mean (SD)
Sex			
Men	648 357 (51)	608 302 (52)	40 055 (43)
Women	620 411 (49)	566 707 (48)	53 704 (57)
Year of birth	1975 (6.15)	1975 (6.11)	1980 (5.02)
Employment status			
Outside labour force	153 481 (12)	124 821 (11)	28 660 (31)
Unemployed	116 532 (9)	100 123 (9)	16 409 (18)
Employed	998 755 (79)	950 065 (81)	48 690 (52)
Education			
Primary	156 189 (12)	132 995 (11)	23 194 (25)
Secondary	588 948 (46)	539 550 (46)	49 398 (53)
High	523 631 (41)	502 464 (43)	21 167 (23)
Personal total income			
Quintile 1 (Q1)	254 913 (20)	212 532 (18)	42 381 (45)
Quintile 2 (Q2)	254 271 (20)	232 540 (20)	21 731 (23)
Quintile 3 (Q3)	254 143 (20)	239 972 (20)	14 171 (15)
Quintile 4 (Q4)	253 251 (20)	243 548 (21)	9703 (10)
Quintile 5 (Q5)	252 190 (20)	246 417 (21)	5773 (6)
Urbanicity			
Missing	13 507 (1)	10 524 (1)	2983 (3)
Urban	816 615 (64)	754 465 (64)	62 150 (66)
Semi-urban	190 802 (15)	178 061 (15)	12 741 (14)
Rural	247 844 (20)	231 959 (20)	15 885 (17)
Cohabitation status			
Living alone	784 571 (62)	714 991 (61)	69 580 (74)
Cohabiting	484 197 (38)	460 018 (39)	24 179 (26)
History of mental disorders			
No	1 175 009 (93)	–	–
Yes	93 759 (7)	–	–
Mental disorder diagnosed over the follow-up			
Any mental disorder	331 657 (26)	259 393 (22)	72 264 (77)
Substance misuse disorders	29 308 (2)	27 222 (2)	2086 (2)
Schizophrenia spectrum disorders	13 057 (1)	9053 (1)	4004 (4)
Mood disorders	140 083 (11)	118 496 (10)	21 587 (23)
Anxiety disorders	160 194 (13)	145 761 (12)	14 433 (15)

The hazard ratios for the association between socioeconomic measures at the age of 30 with the later risk of a mental health disorder are shown in table 2. Lower SEP was consistently associated with a higher risk of being diagnosed with a mental disorder. For example, compared with persons who were employed, being outside the labour force or unemployed were both associated with a twofold risk of a later mental disorder diagnosis. The association between personal total income with a later risk of mental disorder followed a non-linear pattern where the greatest risk for a later mental disorder was observed in the lowest income quintile (HR 2.45, 95% CI 2.42 to 2.48) compared with the highest income quintile. Diagnosis-specific analyses showed that the associations were considerably stronger when substance misuse disorders or schizophrenia spectrum disorders were examined as an outcome; being in the lowest quintile of personal total income was associated with a 4.9-fold risk of later substance misuse disorder and a 5.5-fold risk of later schizophrenia spectrum disorder compared with those in the highest income quintile. Sibling analyses suggested that the associations were diluted, but that the risk remained largely similar when unobserved familial confounders were accounted for (table 2). When the prior history of mental disorders was accounted for, the associations between socioeconomic measures at the age of 30 and later risk of mental disorders were diluted, but the risk patterns remained essentially similar (table 3). Results from the sensitivity analyses showed that the associations were somewhat stronger when diagnoses were retrieved only from the FCR (online supplemental table 1 and 2). When diagnoses were retrieved only from the SAR, the associations were somewhat weaker and there was no clear gradient when personal total income was used as an exposure (online supplemental table 3 and 4).

10.1136/jech-2022-219674.supp1 Supplementary data

**Table 2:** Associations between socioeconomic measures at the age of 30 and later risk of being diagnosed with a mental disorder

	Any mental disorder		F10–19 substance misuse disorders		F20–F29 schizophrenia spectrum disorders		F30–39 mood disorders		F40–F48 anxiety disorders	
	Model A	Model B	Model A	Model B	Model A	Model B	Model A	Model B	Model A	Model B
Employment status										
Outside labour force	1.99 (1.97 to 2.01)	1.82 (1.78 to 1.85)	1.95 (1.88 to 2.02)	1.55 (1.44 to 1.67)	2.96 (2.81 to 3.13)	2.16 (1.94 to 2.40)	1.59 (1.56 to 1.62)	1.37 (1.31 to 1.42)	1.37 (1.35 to 1.4)	1.22 (1.18 to 1.27)
Unemployed	2.07 (2.05 to 2.09)	1.72 (1.68 to 1.76)	3.73 (3.63 to 3.84)	2.35 (2.19 to 2.53)	3.20 (3.03 to 3.38)	2.45 (2.18 to 2.76)	2.07 (2.03 to 2.11)	1.75 (1.68 to 1.83)	1.82 (1.79 to 1.85)	1.51 (1.45 to 1.57)
Employed	ref	ref	ref	ref	ref	ref	ref	ref	ref	ref
Education										
Primary	2.46 (2.44 to 2.49)	2.06 (2.01 to 2.12)	6.60 (6.36 to 6.86)	4.02 (3.66 to 4.42)	2.00 (1.88 to 2.13)	1.66 (1.45 to 1.92)	2.37 (2.32 to 2.42)	1.77 (1.68 to 1.86)	1.93 (1.89 to 1.97)	1.44 (1.37 to 1.51)
Secondary	1.49 (1.48 to 1.50)	1.40 (1.38 to 1.43)	2.87 (2.77 to 2.97)	2.30 (2.12 to 2.48)	1.50 (1.43 to 1.58)	1.41 (1.28 to 1.56)	1.62 (1.60 to 1.65)	1.46 (1.41 to 1.51)	1.39 (1.37 to 1.41)	1.22 (1.18 to 1.26)
High	ref	ref	ref	ref	ref	ref	ref	ref	ref	ref
Personal total income										
Quintile 1	3.07 (3.03 to 3.11)	2.61 (2.54 to 2.68)	4.56 (4.38 to 4.76)	3.16 (2.87 to 3.48)	5.22 (4.83 to 5.65)	4.05 (3.48 to 4.70)	3.15 (3.06 to 3.24)	2.53 (2.40 to 2.67)	2.43 (2.37 to 2.48)	2.00 (1.91 to 2.10)
Quintile 2	2.10 (2.07 to 2.13)	1.89 (1.84 to 1.94)	2.78 (2.66 to 2.90)	2.15 (1.95 to 2.37)	2.67 (2.46 to 2.91)	2.47 (2.12 to 2.88)	2.50 (2.43 to 2.57)	2.23 (2.11 to 2.35)	2.08 (2.03 to 2.13)	1.84 (1.75 to 1.93)
Quintile 3	1.68 (1.66 to 1.71)	1.59 (1.55 to 1.63)	1.87 (1.78 to 1.96)	1.61 (1.46 to 1.78)	1.77 (1.62 to 1.94)	1.77 (1.51 to 2.07)	1.89 (1.84 to 1.95)	1.80 (1.71 to 1.90)	1.70 (1.66 to 1.74)	1.60 (1.52 to 1.68)
Quintile 4	1.38 (1.36 to 1.40)	1.32 (1.28 to 1.32)	1.42 (1.36 to 1.49)	1.26 (1.15 to 1.39)	1.25 (1.14 to 1.38)	1.27 (1.08 to 1.49)	1.45 (1.41 to 1.49)	1.42 (1.35 to 1.50)	1.41 (1.37 to 1.44)	1.32 (1.26 to 1.39)
Quintile 5	ref	ref	ref	ref	ref	ref	ref	ref	ref	ref

Values are hazard ratios and 95% confidence intervals.

Model A: full cohort. Model B: sibling analysis.

**Table 3:** Associations between socioeconomic measures at the age of 30 and later risk of being diagnosed with a mental disorder after adjusting for prior history of a mental disorder

	Any mental disorder		F10–19 substance misuse disorders		F20–F29 Schizophrenia spectrum disorders		F30–39 mood disorders		F40–F48 anxiety disorders	
	Model C	Model D	Model C	Model D	Model C	Model D	Model C	Model D	Model C	Model D
Employment status										
Outside labour force	1.66 (1.64 to 1.67)	1.38 (1.35 to 1.41)	2.09 (2.02 to 2.17)	1.66 (1.54 to 1.79)	3.13 (2.96 to 3.30)	2.38 (2.14 to 2.66)	1.60 (1.57 to 1.64)	1.40 (1.34 to 1.45)	1.43 (1.40 to 1.45)	1.27 (1.22 to 1.32)
Unemployed	1.75 (1.73 to 1.77)	1.48 (1.45 to 1.52)	3.87 (3.76 to 3.98)	2.44 (2.27 to 2.62)	3.29 (3.12 to 3.47)	2.55 (2.27 to 2.88)	2.08 (2.04 to 2.12)	1.78 (1.70 to 1.86)	1.87 (1.84 to 1.91)	1.55 (1.48 to 1.62)
Employed	ref	ref	ref	ref	ref	ref	ref	ref	ref	ref
Education										
Low	1.91 (1.89 to 1.93)	1.64 (1.59 to 1.68)	6.91 (6.65 to 7.17)	4.29 (3.9 to 4.73)	2.03 (1.91 to 2.17)	1.74 (1.51 to 2.01)	2.39 (2.34 to 2.44)	1.80 (1.72 to 1.90)	2.01 (1.97 to 2.05)	1.51 (1.43 to 1.58)
Middle	1.34 (1.33 to 1.35)	1.25 (1.23 to 1.28)	2.91 (2.81 to 3.02)	2.38 (2.2 to 2.58)	1.51 (1.44 to 1.59)	1.45 (1.31 to 1.60)	1.63 (1.60 to 1.66)	1.47 (1.42 to 1.53)	1.41 (1.39 to 1.43)	1.24 (1.21 to 1.29)
High	ref	ref	ref	ref	ref	ref	ref	ref	ref	ref
Personal total income										
Quintile 1	2.45 (2.42 to 2.48)	1.95 (1.90 to 2.00)	4.87 (4.67 to 5.08)	3.38 (3.07 to 3.72)	5.49 (5.07 to 5.94)	4.43 (3.80 to 5.15)	3.20 (3.11 to 3.28)	2.60 (2.46 to 2.74)	2.54 (2.48 to 2.60)	2.10 (2.00 to 2.20)
Quintile 2	1.85 (1.82 to 1.87)	1.63 (1.59 to 1.67)	2.84 (2.72 to 2.97)	2.23 (2.03 to 2.47)	2.73 (2.50 to 2.97)	2.57 (2.20 to 3.01)	2.52 (2.45 to 2.59)	2.26 (2.15 to 2.39)	2.12 (2.07 to 2.17)	1.89 (1.80 to 1.98)
Quintile 3	1.56 (1.54 to 1.58)	1.47 (1.43 to 1.51)	1.89 (1.80 to 1.98)	1.64 (1.49 to 1.81)	1.79 (1.64 to 1.96)	1.81 (1.55 to 2.12)	1.90 (1.85 to 1.96)	1.82 (1.73 to 1.92)	1.71 (1.67 to 1.76)	1.62 (1.54 to 1.70)
Quintile 4	1.32 (1.31 to 1.34)	1.27 (1.23 to 1.30)	1.43 (1.36 to 1.50)	1.27 (1.15 to 1.40)	1.26 (1.14 to 1.38)	1.29 (1.09 to 1.51)	1.45 (1.41 to 1.50)	1.43 (1.35 to 1.51)	1.41 (1.38 to 1.45)	1.33 (1.27 to 1.40)
Quintile 5	ref	ref	ref	ref	ref	ref	ref	ref	ref	ref

Values are hazard ratios and 95% confidence intervals. Analyses are further adjusted for the prior history of a mental disorder.

Model C: full cohort. Model D: sibling analysis.

**Figure 1:**
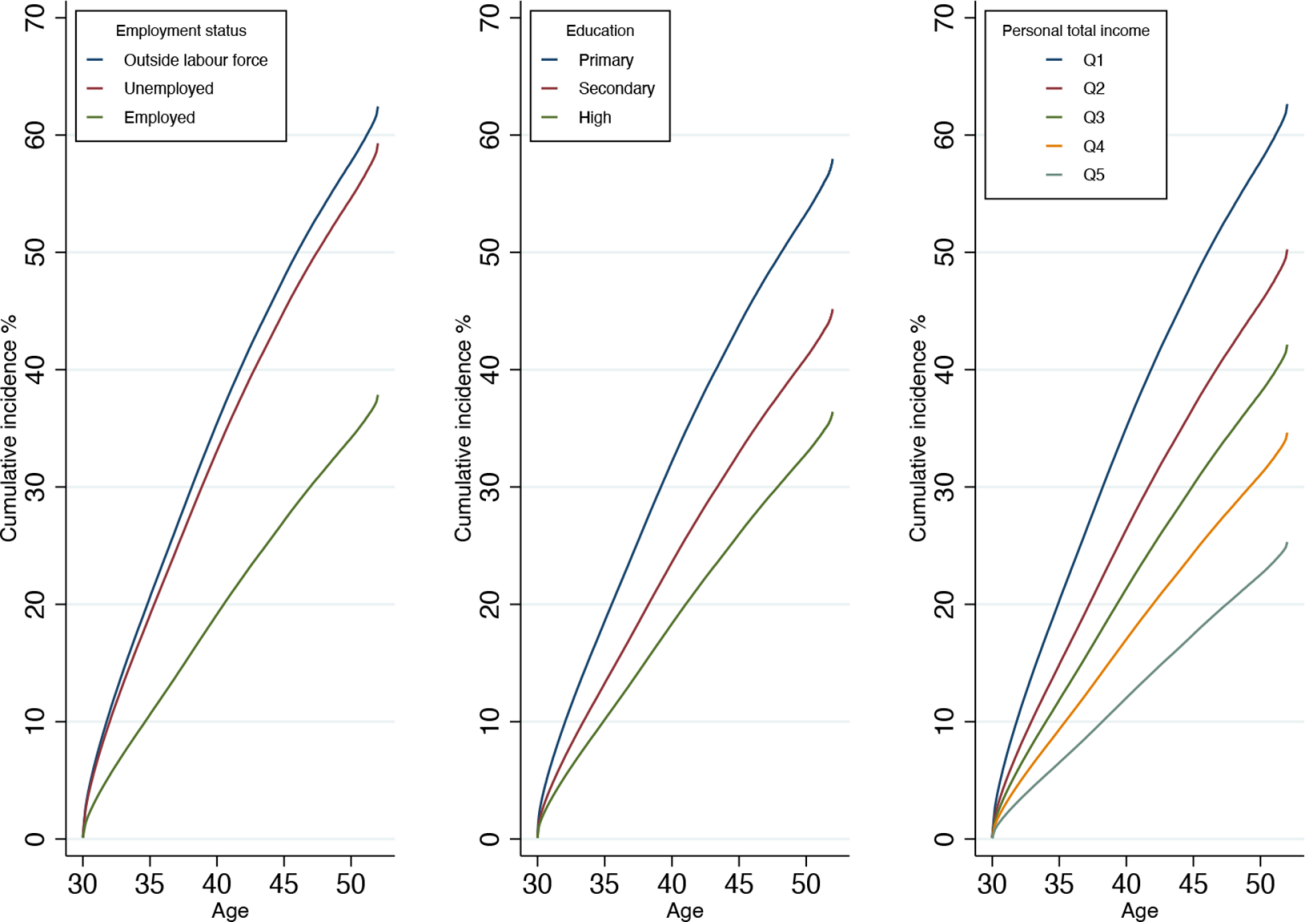
Cumulative incidence of any mental disorder across levels of three socioeconomic measures (employment status, education and personal total income) assessed at age 30. Q, quintile.

The cumulative incidences—that is, absolute risk estimates of being diagnosed with any mental disorder by the age of 52—across different categories of SEP are shown in figure 1. Over the study follow-up, 38% of those who were employed at the age of 30 were later diagnosed with a mental disorder by the age of 52. The corresponding estimates for those who were unemployed or outside the labour force were 59% and 62%, respectively. Of the persons who had completed only primary education, 58% were diagnosed with a mental disorder during the follow-up period. The corresponding estimates for those persons who had completed secondary or higher education were 45% and 36%, respectively. Lastly, 63% of those in the lowest quintile of personal income at the age of 30 were diagnosed with a mental disorder during the follow-up, whereas the corresponding figure for those in the highest quintile of personal income was 25%.

The diagnosis-specific cumulative incidence estimates are shown in table 4. The lowest absolute risk of being diagnosed with a specific mental disorder was consistently found among those persons who were employed, had high education or high personal total income at the age of 30. However, notable differences in the estimates across diagnostic categories were observed. For example, among persons who were employed at the age of 30, the absolute risk of being diagnosed with a substance misuse disorder or schizophrenia spectrum disorder was 4% and 1%, respectively. On the other hand, the absolute risk estimates of being diagnosed with mood disorder or anxiety disorder were 19% and 24%, respectively. Across quintiles of personal total income, the absolute risk estimates of being diagnosed with a substance misuse or schizophrenia spectrum disorder by the age of 52 were disproportionately higher in the lowest quintile (Q1), while a similar trend was not observed with respect to mood or anxiety disorders.

**Table 4:** Diagnosis-specific cumulative incidence estimates up to age 52 across levels of different socioeconomic measures at the age of 30

	Substance misuse disorders	Schizophrenia spectrum disorders	Mood disorders	Anxiety disorders
Employment status				
Outside labour force	12	11	32	36
Unemployed	19	5	32	36
Employed	4	1	19	24
Education				
Primary	19	6	30	33
Secondary	7	3	23	28
High	2	1	17	24
Personal total income				
Quintile 1	15	8	33	37
Quintile 2	6	2	27	32
Quintile 3	4	1	21	27
Quintile 4	4	1	16	22
Quintile 5	3	1	11	12

Values indicate the absolute risk (in percentages) of being diagnosed with the disorder of interest by age 52.

The cumulative incidence estimates for those persons without a prior history of mental disorder are shown in online supplemental table 5. When compared with the whole study population, the cumulative incidence estimates of any mental disorder were up to 12% points lower and for specific diagnoses up to 8% points lower. The estimates were especially lower among those persons who were either unemployed or outside the labour force or had low education or low personal total income at the age of 30.

## Discussion

Based on a nationwide cohort study of more than 1.2 million Finnish persons, those who had low educational attainment, who were outside the labour force or unemployed, or who had low personal income at age 30 years were at an increased risk of being later diagnosed with a mental disorder. The association of low SEP with a later risk of mental disorder was significantly stronger for substance misuse and schizophrenia spectrum disorders than for mood and anxiety disorders. An important finding was that shared family characteristics, potentially influencing SEP as well as the incidence of mental disorders, and prior history of mental disorders only partially explained the association between SEP at the age of 30 and the later risk of mental disorders.

The findings of the present study are in accordance with the literature where low SEP has been linked to poor mental health.^1–10^ The association between SEP and mental health has been observed at different life stages, both among children and adolescents growing up in low SEP households as well as in adults. Where most previous studies have focused only on educational attainment or occupational status as the primary measure of SEP, in this study we used three different measures of SEP including personal total income that measures directly available economic resources. Although these measures of SEP are correlated, they represent different facets of social stratification and may thus be differently relevant to mental disorders.^28^ The present findings consistently show that, regardless of which measure is used, poor SEP at the age of 30 is significantly associated with a later risk of mental disorders across a spectrum of different mental disorders, but most strongly with substance misuse and schizophrenia spectrum disorders.

The absolute risks of being diagnosed with a later mental disorder among persons who had low SEP at the age of 30 years were considerable. For example, 58% of those who had low educational attainment at the age of 30 were diagnosed with a mental disorder by the age of 52; in comparison, the corresponding estimate for those with high educational attainment was 36%. These findings show that poor SEP is an important risk factor and predictor of mental health disorders over the life course from adulthood to middle age. They also echo recent findings which highlight the high prevalence of mental disorders over the life course,^29^ and thus show how mental disorders affect a considerable proportion of the population.

Traditionally, the association between SEP and mental health has been explained by both social causation (ie, poor socioeconomic conditions causing poor mental health) and health selection (ie, poor mental health leading to poor SEP).^30–32^ Although our study was not explicitly designed to examine and compare these two hypotheses, the present findings may provide support for both social causation and health selection. Those persons in the study population who had been diagnosed with a mental disorder before the age of 30 had a considerably lower rate of employment, higher education and high income. This result is consistent with prior findings where mental disorders diagnosed in adolescence and early adulthood are associated with poor socioeconomic prospects in the long run.^11–14^ On the contrary, the greater risk of mental disorders observed among persons with lower SEP support the importance of social causality—that is, adverse social conditions causing poor mental health. From the different mechanisms, psychological stress of living with limited economic resources, poor physical health associated with poverty and poorer health behaviours among people with lower SEP are likely important.^7^

### Strengths and limitations

The use of a nationwide study population with complete follow-up, sibling design and both secondary and primary care psychiatric register data are the main strengths of the present study. However, when interpreting the present findings, there are some limitations that need to be considered. First, the registers covered long-term sickness absences and primary care only starting from 2005 and 2011, respectively. Thus, it is likely that mental disorders of persons with milder symptoms, who were only treated by a general practitioner before the year 2005 or who did not seek help from any type of healthcare service for their mental disorders, were not included. Because of this, it is also likely that the information of mental disorders before the age of 30 is an underestimate of the underlying prevalence of these conditions. Furthermore, although register-based mental disorder diagnoses in the FCR have been shown to have good validity,^33^ not all data on mental disorders in the FCR have been validated. In addition, it is unclear whether some diagnoses registered in the SAR describe more acute stress responses to difficult situations than actual mental disorders. Detailed information of psychosocial factors which could help to understand the association between SEP and mental disorders were not recorded in the registers used in the present study. Thus, the possibility of unmeasured or residual confounding cannot be ruled out in observational studies such as ours, and the results should be interpreted with appropriate caution. Last, Finland’s healthcare system is subsidised by the government providing universal access to healthcare services for all citizens. Economic resources of unemployed job seekers are also strongly subsidised through unemployment benefits. Therefore, the present findings might not be generalisable to other developed countries with different institutional settings.

## Conclusions

Our results, based on a nationwide cohort of 1.2 million Finnish persons, showed an association of the SEP at the age of 30 with the later risk of mental disorders. These findings suggest that the burden of mental disorders is vastly greater among persons with low SEP, and policies enhancing social mobility or allocating greater amounts of preventive measures to persons with low SEP could mitigate the disease burden of mental disorders in the society.

## Data Availability

Data may be obtained from a third party and are not publicly available. The data that support the findings of this study are available from the Finnish Institute for Health and Welfare, Social Insurance Institution of Finland, and Statistics Finland. Restrictions apply to the availability of these data, which were used under license for this study. For information on accessing the data see www.thl.fi, www.kela.fi, and www.stat.fi.

## References

[1] Suokas K , Koivisto A-M , Hakulinen C , et al . Association of income with the incidence rates of first psychiatric hospital admissions in Finland, 1996-2014. JAMA Psychiatry 2020;77:274–84. 10.1001/jamapsychiatry.2019.3647 31851325 PMC6990744

[2] Muntaner C , Eaton WW , Miech R , et al . Socioeconomic position and major mental disorders. Epidemiol Rev 2004;26:53–62. 10.1093/epirev/mxh001 15234947

[3] Sareen J , Afifi TO , McMillan KA , et al . Relationship between household income and mental disorders. Arch Gen Psychiatry 2011;68:419. 10.1001/archgenpsychiatry.2011.15 21464366

[4] Fryers T , Melzer D , Jenkins R . Social inequalities and the common mental disorders: a systematic review of the evidence. Soc Psychiatry Psychiatr Epidemiol 2003;38:229–37. 10.1007/s00127-003-0627-2 12719837

[5] Reiss F . Socioeconomic inequalities and mental health problems in children and adolescents: a systematic review. Soc Sci Med 2013;90:24–31. 10.1016/j.socscimed.2013.04.026 23746605

[6] Commission on Social Determinants of Health . Closing the gap in a generation: health equity through action on the social determinants of health. World Health Organization, 2008. 10.1016/S0140-6736(08)61690-6 18994664

[7] Ridley M , Rao G , Schilbach F , et al . Poverty, depression, and anxiety: causal evidence and mechanisms. Science 2020;370:eaay0214. 10.1126/science.aay0214 33303583

[8] Gilman SE , Kawachi I , Fitzmaurice GM , et al . Socioeconomic status in childhood and the lifetime risk of major depression. Int J Epidemiol 2002;31:359–67. 10.1093/ije/31.2.359 11980797

[9] Hakulinen C , Webb RT , Pedersen CB , et al . Parental income during childhood and later risk of developing schizophrenia: a national cohort study. JAMA Psychiatry 2020;77:17–24. 10.1001/jamapsychiatry.2019.2299 31642886 PMC6813592

[10] Hakulinen C , Mok PLH , Horsdal HT , et al . Parental income as a marker for socioeconomic position during childhood and later risk of developing a secondary care-diagnosed mental disorder examined across the full diagnostic spectrum: a national cohort study. BMC Med 2020;18:323. 10.1186/s12916-020-01794-5 33190641 PMC7667856

[11] Hakulinen C , Musliner KL , Agerbo E . Bipolar disorder and depression in early adulthood and long-term employment, income, and educational attainment: a nationwide cohort study of 2,390,127 individuals. Depress Anxiety 2019;36:1080–8. 10.1002/da.22956 31508865

[12] Hakulinen C , McGrath JJ , Timmerman A , et al . The association between early-onset schizophrenia with employment, income, education, and cohabitation status: nationwide study with 35 years of follow-up. Soc Psychiatry Psychiatr Epidemiol 2019;54:1343–51. 10.1007/s00127-019-01756-0 31456027

[13] Hakulinen C , Elovainio M , Arffman M , et al . Mental disorders and long-term labour market outcomes: nationwide cohort study of 2 055 720 individuals. Acta Psychiatr Scand 2019;140:371–81. 10.1111/acps.13067 31254386

[14] Linder A , Gerdtham U-G , Trygg N , et al . Inequalities in the economic consequences of depression and anxiety in Europe: a systematic scoping review. Eur J Public Health 2020;30:767–77. 10.1093/eurpub/ckz127 31302703 PMC7445046

[15] Marwaha S , Durrani A , Singh S . Employment outcomes in people with bipolar disorder: a systematic review. Acta Psychiatr Scand 2013;128:179–93. 10.1111/acps.12087 23379960

[16] Hakulinen C , Elovainio M , Arffman M , et al . Employment status and personal income before and after onset of a severe mental disorder: a case-control study. Psychiatr Serv 2020;71:250–5. 10.1176/appi.ps.201900239 31722646

[17] Marwaha S , Johnson S . Schizophrenia and employment - a review. Soc Psychiatry Psychiatr Epidemiol 2004;39:337–49. 10.1007/s00127-004-0762-4 15133589

[18] Davidson M , Kapara O , Goldberg S , et al . A nation-wide study on the percentage of schizophrenia and bipolar disorder patients who earn minimum wage or above. Schizophr Bull 2016;42:443–7. 10.1093/schbul/sbv023 25796051 PMC4753584

[19] Hakulinen C , Böckerman P , Pulkki-Råback L , et al . Employment and earnings trajectories before and after sickness absence due to major depressive disorder: a nationwide case–control study. Occup Environ Med 2021;78:173–8. 10.1136/oemed-2020-106660 33051385

[20] Mojtabai R , Stuart EA , Hwang I , et al . Long-term effects of mental disorders on employment in the National Comorbidity Survey ten-year follow-up. Soc Psychiatry Psychiatr Epidemiol 2015;50:1657–68. 10.1007/s00127-015-1097-z 26211661 PMC4618045

[21] Galobardes B , Shaw M , Lawlor DA , et al . Indicators of socioeconomic position (part 1). J Epidemiol Community Health 2006;60:7–12. 10.1136/jech.2004.023531 PMC2465546 16361448

[22] Lahelma E , Laaksonen M , Martikainen P , et al . Multiple measures of socioeconomic circumstances and common mental disorders. Soc Sci Med 2006;63:1383–99. 10.1016/j.socscimed.2006.03.027 16690186

[23] Spiegelhalter D . Risk and uncertainty communication. Annu Rev Stat Appl 2017;4:31–60. 10.1146/annurev-statistics-010814-020148

[24] WONCA International Classification Committee (WICC) . ICPC-2-R: International classification of primary care. New York: Oxford University Press, 2005.

[25] Suokas K . hilmo_identify_episodes V 1.1.1; 2021. 10.5281/zenodo.5381082

[26] Sjölander A , Frisell T , Öberg S . Sibling comparison studies. Annu Rev Stat Appl 2022;9:71–94. 10.1146/annurev-statistics-040120-024521

[27] Andersen PK , Geskus RB , de Witte T , et al . Competing risks in epidemiology: possibilities and pitfalls. Int J Epidemiol 2012;41:861–70. 10.1093/ije/dyr213 22253319 PMC3396320

[28] Galobardes B , Lynch J , Smith GD . Measuring socioeconomic position in health research. Br Med Bull 2007;81-82:21–37. 10.1093/bmb/ldm001 17284541

[29] Caspi A , Houts RM , Ambler A , et al . Longitudinal assessment of mental health disorders and comorbidities across 4 decades among participants in the Dunedin Birth Cohort Study. JAMA Netw Open 2020;3:e203221. 10.1001/jamanetworkopen.2020.3221 32315069 PMC7175086

[30] Dohrenwend BP , Levav I , Shrout PE , et al . Socioeconomic status and psychiatric disorders: the causation-selection issue. Science 1992;255:946–52. 10.1126/science.1546291 1546291

[31] Hudson CG . Socioeconomic status and mental illness: tests of the social causation and selection hypotheses. Am J Orthopsychiatry 2005;75:3–18. 10.1037/0002-9432.75.1.3 15709846

[32] Miech RA , Caspi A , Moffitt TE , et al . Low socioeconomic status and mental disorders: a longitudinal study of selection and causation during young adulthood. Am J Sociol 1999;104:1096–131. 10.1086/210137

[33] Sund R . Quality of the Finnish hospital discharge register: a systematic review. Scand J Public Health 2012;40:505–15. 10.1177/1403494812456637 22899561

