# Supplementary material for: Socioeconomic position at the age of 30 and the later risk of a mental disorder: a nationwide population-based register study
Source: J Epidemiol Community Health. 2023 Feb 6;77(5):298–304. doi: 10.1136/jech-2022-219674 (PMC10086472; doi:10.1136/jech-2022-219674)
Supplement: Supplementary data [file jech-2022-219674supp001.pdf]

Online Supplemental Appendix 1. The associations between socioeconomic measures at the age of 30 and later risk of being diagnosed with a mental disorder diagnosis retrieved from the Finnish Care Register for Health Care (FCR)

	Any mental disorder		F10-19 Substance misuse disorders		F20-F29 Schizophrenia spectrum disorders		F30-39 Mood disorders		F40-F48 Anxiety disorders	
	Model A	Model B	Model A	Model B	Model A	Model B	Model A	Model B	Model A	Model B
**Employment status**										
Outside labour force	2.55 (2.53-2.58)	2.35 (2.30-2.40)	3.79 (3.70-3.89)	2.85 (2.69-3.02)	11.13 (10.8-11.47)	7.67 (7.15-8.23)	2.39 (2.36-2.43)	2.04 (1.97-2.10)	1.94 (1.91-1.97)	1.71 (1.66-1.76)
Unemployed	2.55 (2.52-2.58)	2.13 (2.07-2.18)	5.14 (5.03-5.26)	3.20 (3.02-3.39)	3.98 (3.82-4.13)	3.01 (2.77-3.28)	2.56 (2.51-2.60)	2.11 (2.03-2.19)	2.30 (2.26-2.33)	1.89 (1.82-1.96)
Employed	ref.	ref.	ref.	ref.	ref.	ref.	ref.	ref.	ref.	ref.
**Education**										
Primary	3.02 (2.99-3.06)	2.54 (2.46-2.61)	9.95 (9.63-10.28)	6.26 (5.77-6.79)	4.59 (4.42-4.78)	4.85 (4.43-5.30)	3.10 (3.04-3.15)	2.37 (2.27-2.48)	2.60 (2.56-2.64)	1.96 (1.88-2.04)
Secondary	1.66 (1.64-1.67)	1.56 (1.53-1.59)	3.47 (3.36-3.58)	2.82 (2.63-3.02)	2.31 (2.23-2.39)	2.52 (2.36-2.70)	1.86 (1.84-1.89)	1.68 (1.63-1.74)	1.57 (1.55-1.59)	1.39 (1.35-1.43)
High	ref.	ref.	ref.	ref.	ref.	ref.	ref.	ref.	ref.	ref.
**Personal total income**										
Q1	4.49 (4.42-4.56)	3.90 (3.78-4.02)	7.89 (7.6-8.19)	5.58 (5.13-6.07)	15.34 (14.38-16.36)	13.17 (11.68-14.86)	4.71 (4.60-4.83)	3.79 (3.62-3.98)	3.59 (3.51-3.66)	2.95 (2.82-3.08)
Q2	2.69 (2.65-2.74)	2.48 (2.4-2.55)	3.48 (3.34-3.62)	2.79 (2.56-3.04)	4.74 (4.42-5.08)	4.70 (4.15-5.31)	3.07 (2.99-3.14)	2.77 (2.64-2.90)	2.50 (2.44-2.56)	2.22 (2.12-2.32)
Q3	1.93 (1.90-1.96)	1.83 (1.78-1.89)	2.05 (1.96-2.14)	1.83 (1.67-2.00)	2.16 (2.00-2.33)	2.35 (2.07-2.67)	2.08 (2.03-2.14)	1.99 (1.89-2.09)	1.87 (1.83-1.91)	1.73 (1.66-1.82)
Q4	1.48 (1.46-1.51)	1.42 (1.38-1.47)	1.47 (1.40-1.54)	1.33 (1.22-1.45)	1.34 (1.24-1.46)	1.45 (1.27-1.66)	1.51 (1.47-1.56)	1.49 (1.41-1.56)	1.47 (1.44-1.51)	1.39 (1.33-1.46)
Q5	ref.	ref.	ref.	ref.	ref.	ref.	ref.	ref.	ref.	ref.

Note. Values are hazard ratios and 95% confidence intervals
Model A: full cohort
Model B: sibling analysis

Online Supplemental Appendix 2. The associations between socioeconomic measures at the age of 30 and later risk of being diagnosed with a mental disorder retrieved from the FCR after adjusting for prior history of a mental disorder

	Any mental disorder		F10-19 Substance misuse disorders		F20-F29 Schizophrenia spectrum disorders		F30-39 Mood disorders		F40-F48 Anxiety disorders	
	Model C	Model D	Model C	Model D	Model C	Model D	Model C	Model D	Model C	Model D
**Employment status**										
Outside labour force	2.05 (2.03-2.07)	1.74 (1.70-1.79)	2.13 (2.08-2.19)	1.89 (1.77-2.01)	4.72 (4.57-4.87)	3.25 (2.97-3.55)	1.59 (1.56-1.61)	1.51 (1.46-1.57)	1.45 (1.43-1.47)	1.37 (1.33-1.42)
Unemployed	2.08 (2.06-2.10)	1.82 (1.77-1.88)	3.80 (3.71-3.89)	2.56 (2.40-2.73)	2.06 (1.98-2.14)	2.15 (1.94-2.38)	1.94 (1.90-1.97)	1.78 (1.70-1.85)	1.89 (1.86-1.92)	1.64 (1.57-1.70)
Employed	ref.	ref.	ref.	ref.	ref.	ref.	ref.	ref.	ref.	ref.
**Education**										
low	2.23 (2.20-2.26)	1.95 (1.89-2.01)	6.63 (6.41-6.86)	4.49 (4.11-4.90)	1.75 (1.68-1.83)	2.13 (1.89-2.39)	2.04 (2-2.08)	1.82 (1.74-1.91)	1.94 (1.91-1.97)	1.57 (1.51-1.65)
middle	1.45 (1.44-1.46)	1.36 (1.33-1.39)	2.91 (2.82-3.00)	2.37 (2.20-2.55)	1.50 (1.45-1.55)	1.54 (1.41-1.68)	1.58 (1.56-1.6)	1.47 (1.42-1.52)	1.39 (1.37-1.40)	1.25 (1.21-1.29)
high	ref.	ref.	ref.	ref.	ref.	ref.	ref.	ref.	ref.	ref.
**Personal total income**										
Q1	3.43 (3.38-3.48)	2.84 (2.75-2.93)	4.83 (4.65-5.01)	3.75 (3.43-4.10)	5.77 (5.40-6.17)	5.10 (4.44-5.86)	3.06 (2.98-3.14)	2.74 (2.60-2.88)	2.65 (2.59-2.71)	2.29 (2.19-2.40)
Q2	2.31 (2.27-2.34)	2.09 (2.02-2.16)	2.78 (2.67-2.89)	2.26 (2.06-2.48)	2.78 (2.59-2.98)	2.66 (2.31-3.07)	2.49 (2.43-2.56)	2.31 (2.19-2.43)	2.16 (2.11-2.21)	1.94 (1.85-2.03)
Q3	1.75 (1.72-1.77)	1.67 (1.61-1.72)	1.85 (1.77-1.93)	1.65 (1.50-1.82)	1.62 (1.50-1.74)	1.81 (1.56-2.10)	1.85 (1.8-1.9)	1.80 (1.70-1.89)	1.72 (1.68-1.76)	1.61 (1.54-1.69)
Q4	1.41 (1.38-1.43)	1.36 (1.31-1.40)	1.40 (1.34-1.47)	1.29 (1.17-1.41)	1.17 (1.08-1.27)	1.30 (1.11-1.51)	1.43 (1.39-1.47)	1.42 (1.35-1.50)	1.41 (1.38-1.45)	1.33 (1.27-1.40)
Q5	ref.	ref.	ref.	ref.	ref.	ref.	ref.	ref.	ref.	ref.

Note. Values are hazard ratios and 95% confidence intervals. Analyses are further adjusted for the prior history of a mental disorder.
Model C: full cohort
Model D: sibling analysis

Online Supplemental Appendix 3. The associations between socioeconomic measures at the age of 30 and later risk of being diagnosed with a mental disorder diagnosis retrieved from the sickness absence register (SAR)

	Any mental disorder		F10-19 Substance misuse disorders		F20-F29 Schizophrenia spectrum disorders		F30-39 Mood disorders		F40-F48 Anxiety disorders	
	Model A	Model B	Model A	Model B	Model A	Model B	Model A	Model B	Model A	Model B
**Employment status**										
Outside labour force	1.13 (1.12-1.15)	0.98 (0.96-1.01)	3.79 (3.7-3.89)	2.85 (2.69-3.02)	11.13 (10.8-11.47)	7.67 (7.15-8.23)	2.39 (2.36-2.43)	2.04 (1.97-2.10)	1.94 (1.91-1.97)	1.71 (1.66-1.76)
Unemployed	1.59 (1.57-1.61)	1.32 (1.28-1.36)	5.14 (5.03-5.26)	3.20 (3.02-3.39)	3.98 (3.82-4.13)	3.01 (2.77-3.28)	2.56 (2.51-2.60)	2.11 (2.03-2.19)	2.30 (2.26-2.33)	1.89 (1.82-1.96)
Employed	ref.	ref.	ref.	ref.	ref.	ref.	ref.	ref.	ref.	ref.
**Education**										
Primary	1.76 (1.74-1.79)	1.4 (1.36-1.45)	9.95 (9.63-10.28)	6.26 (5.77-6.79)	4.59 (4.42-4.78)	4.85 (4.43-5.3)	3.10 (3.04-3.15)	2.37 (2.27-2.48)	2.60 (2.56-2.64)	1.96 (1.88-2.04)
Secondary	1.35 (1.34-1.36)	1.24 (1.21-1.27)	3.47 (3.36-3.58)	2.82 (2.63-3.02)	2.31 (2.23-2.39)	2.52 (2.36-2.7)	1.86 (1.84-1.89)	1.68 (1.63-1.74)	1.57 (1.55-1.59)	1.39 (1.35-1.43)
High	ref.	ref.	ref.	ref.	ref.	ref.	ref.	ref.	ref.	ref.
**Personal total income**										
Q1	1.78 (1.75-1.81)	1.43 (1.39-1.48)	7.89 (7.6-8.19)	5.58 (5.13-6.07)	15.34 (14.38-16.36)	13.17 (11.68-14.86)	4.71 (4.60-4.83)	3.79 (3.62-3.98)	3.59 (3.51-3.66)	2.95 (2.82-3.08)
Q2	1.67 (1.64-1.7)	1.46 (1.41-1.5)	3.48 (3.34-3.62)	2.79 (2.56-3.04)	4.74 (4.42-5.08)	4.70 (4.15-5.31)	3.07 (2.99-3.14)	2.77 (2.64-2.90)	2.50 (2.44-2.56)	2.22 (2.12-2.32)
Q3	1.54 (1.51-1.56)	1.43 (1.39-1.48)	2.05 (1.96-2.14)	1.83 (1.67-2.00)	2.16 (2.00-2.33)	2.35 (2.07-2.67)	2.08 (2.03-2.14)	1.99 (1.89-2.09)	1.87 (1.83-1.91)	1.73 (1.66-1.82)
Q4	1.30 (1.28-1.32)	1.23 (1.19-1.27)	1.47 (1.4-1.54)	1.33 (1.22-1.45)	1.34 (1.24-1.46)	1.45 (1.27-1.66)	1.51 (1.47-1.56)	1.49 (1.41-1.56)	1.47 (1.44-1.51)	1.39 (1.33-1.46)
Q5	ref.	ref.	ref.	ref.	ref.	ref.	ref.	ref.	ref.	ref.

Note. Values are hazard ratios and 95% confidence intervals
Model A: full cohort
Model B: sibling analysis

Online Supplemental Appendix 4. The associations between socioeconomic measures at the age of 30 and later risk of being diagnosed with a mental disorder diagnosis retrieved from the SAR after adjusting for prior history of a mental disorder

	Any mental disorder		F10-19 Substance misuse disorders		F20-F29 Schizophrenia spectrum disorders		F30-39 Mood disorders		F40-F48 Anxiety disorders	
	Model C	Model D	Model C	Model D	Model C	Model D	Model C	Model D	Model C	Model D
**Employment status**										
Outside labour force	0.89 (0.88-0.91)	0.84 (0.82-0.86)	2.13 (2.08-2.19)	1.89 (1.77-2.01)	4.72 (4.57-4.87)	3.25 (2.97-3.55)	1.59 (1.56-1.61)	1.51 (1.46-1.57)	1.45 (1.43-1.47)	1.37 (1.33-1.42)
Unemployed	1.38 (1.36-1.40)	1.19 (1.15-1.22)	3.80 (3.71-3.89)	2.56 (2.4-2.73)	2.06 (1.98-2.14)	2.15 (1.94-2.38)	1.94 (1.90-1.97)	1.78 (1.70-1.85)	1.89 (1.86-1.92)	1.64 (1.57-1.70)
Employed	ref.	ref.	ref.	ref.	ref.	ref.	ref.	ref.	ref.	ref.
**Education**										
low	1.43 (1.41-1.45)	1.23 (1.19-1.28)	6.63 (6.41-6.86)	4.49 (4.11-4.90)	1.75 (1.68-1.83)	2.13 (1.89-2.39)	2.04 (2.00-2.08)	1.82 (1.74-1.91)	1.94 (1.91-1.97)	1.57 (1.51-1.65)
middle	1.25 (1.24-1.26)	1.17 (1.14-1.19)	2.91 (2.82-3.00)	2.37 (2.2-2.55)	1.50 (1.45-1.55)	1.54 (1.41-1.68)	1.58 (1.56-1.6)	1.47 (1.42-1.52)	1.39 (1.37-1.40)	1.25 (1.21-1.29)
high	ref.	ref.	ref.	ref.	ref.	ref.	ref.	ref.	ref.	ref.
**Personal total income**										
Q1	1.42 (1.39-1.44)	1.19 (1.15-1.23)	4.83 (4.65-5.01)	3.75 (3.43-4.1)	5.77 (5.4-6.17)	5.10 (4.44-5.86)	3.06 (2.98-3.14)	2.74 (2.6-2.88)	2.65 (2.59-2.71)	2.29 (2.19-2.40)
Q2	1.51 (1.48-1.54)	1.33 (1.28-1.37)	2.78 (2.67-2.89)	2.26 (2.06-2.48)	2.78 (2.59-2.98)	2.66 (2.31-3.07)	2.49 (2.43-2.56)	2.31 (2.19-2.43)	2.16 (2.11-2.21)	1.94 (1.85-2.03)
Q3	1.46 (1.44-1.49)	1.36 (1.32-1.41)	1.85 (1.77-1.93)	1.65 (1.50-1.82)	1.62 (1.50-1.74)	1.81 (1.56-2.10)	1.85 (1.80-1.90)	1.80 (1.70-1.89)	1.72 (1.68-1.76)	1.61 (1.54-1.69)
Q4	1.27 (1.25-1.29)	1.2 (1.16-1.24)	1.40 (1.34-1.47)	1.29 (1.17-1.41)	1.17 (1.08-1.27)	1.30 (1.11-1.51)	1.43 (1.39-1.47)	1.42 (1.35-1.50)	1.41 (1.38-1.45)	1.33 (1.27-1.40)
Q5	ref.	ref.	ref.	ref.	ref.	ref.	ref.	ref.	ref.	ref.

Note. Values are hazard ratios and 95% confidence intervals. Analyses are further adjusted for the prior history of a mental disorder.
Model C: full cohort
Model D: sibling analysis

Online Supplemental Appendix 5. Cumulative incidence estimates up to age 52 across levels of different socioeconomic measures at the age of 30 among persons without a mental disorder before age 30

	Any mental disorder	Substance misuse disorders	Schizophrenia spectrum disorders	Mood disorders	Anxiety disorders
**Employment status**					
Outside labour force	50	6	3	25	30
Unemployed	52	14	3	27	30
Employed	37	4	1	17	23
**Education**					
Primary	49	13	2	25	28
Secondary	41	6	2	20	25
High	36	2	1	16	23
**Personal total income**					
Q1	52	10	3	27	31
Q2	47	5	1	24	30
Q3	41	4	1	20	26
Q4	34	4	1	15	21
Q5	25	3	1	11	15

Note. Values indicate the absolute risk (in percentages) of being diagnosed with the disorder of interest by age 52; Q=quintile
